# Diagnostic dilemmas of squamous differentiation in prostate carcinoma case report and review of the literature

**DOI:** 10.1186/1746-1596-6-46

**Published:** 2011-05-31

**Authors:** Nicoleta C Arva, Kasturi Das

**Affiliations:** 1 Department of Pathology, New York University Medical Center, 550 First Avenue, New York, NY, 10016, USA

## Abstract

We report a case of pure squamous cell carcinoma involving the prostate and urinary bladder and describe the diagnostic dilemmas that we faced in trying to determine its origin. The patient was diagnosed ten years ago with prostatic adenocarcinoma treated with radioactive seed implantation. During the last year he also underwent a TURP procedure for urinary obstruction complicated by multiple infections. Postsurgery, the patient developed colo-urethral fistula and decision to perform cystprostatectomy was taken. Excision illustrated a tumor mass replacing the entire prostate that microscopically proved to be squamous cell carcinoma. The challenge that we encountered was to determine its origin, the possibilities being divergent differentiation from adenocarcinoma post radiation therapy, de novo neoplasm or urothelial carcinoma with extensive squamous differentiation. Our literature review showed also that the etiology of prostatic squamous carcinoma is still unclear. We present our approach in an attempt to solve this dilemma.

## Background

Squamous cell carcinoma of the prostate is a rare entity, representing less than 1% of all prostate carcinomas. About half of the cases arise after endocrine or radiation therapy for adenocarcinoma. However, cases occurring "de novo", in patients with no history of prostatic disease have also been reported. This finding points toward multiple etiologies for this disease. It is hypothesized that squamous differentiation in prostate cancer has an origin in the urothelial lining of the prostatic urethra or periurethral ducts. It is also speculated that it can be derived from pluripotent stem cells capable of multidirectional differentiation.

Morphologically, squamous differentiation in prostate cancer can be encountered in pure form or associated with adenocarcinoma, urothelial carcinoma or sarcoma. Given its multiple possible origins, a decision as to whether the squamous component develops through divergent differentiation from adenocarcinoma following treatment, represents squamous differentiation of a transitional cell carcinoma, or is a pure second prostatic malignancy can be very challenging.

Our literature review shows that the etiology of squamous differentiation in prostate cancer is not well defined. Understanding the biology of this tumor might help to develop more efficient therapies for this aggressive malignancy with a poor prognosis. We describe our findings and approach of this case to help pathologists encountering this unusual prostatic neoplasm.

## Case Presentation

The patient is a 77 year old male with a history of prostate cancer for which he was treated with brachytherapy (radiation seeds) 10 years ago. Patient had also a transurethral resection of prostate due to urinary obstruction complicated by multiple tract infections one year prior to his current presentation. At present, patient was admitted for decreased urinary output. Laboratory work up revealed a creatinin level of 10 mg/dl, with metabolic acidosis, thought to be secondary to acute kidney injury. Computer tomography imaging studies showed bilateral hydronephrosis grade 2, with obstruction of the ureters at the uretero-vesicle junction for which percutaneous nephrostomy tubes were placed. A cystourethrogram was also performed and revealed colo-urethral fistula in the region of the prostatic urethra, extending to the rectum. The bladder lumen was irregular and small, corresponding to thickened bladder wall seen on CT. Patient was taken to surgery for cystprostatectomy, ileal conduit and repair of the recto- urethral fistula.

The cystprostatectomy specimen was composed of a portion of prostate (4.2 × 2.7 × 2.5 cm), urinary bladder (7.5 × 5 cm) and the surrounding fat. The entire prostatic gland was replaced by a white firm irregular tumor mass. The bladder mucosa was very congested, diffusely irregular but no mucosal growths or luminal masses were present. The thickness of the bladder wall ranged from 0.5 to 1.0 cm.

**Figure 1:**
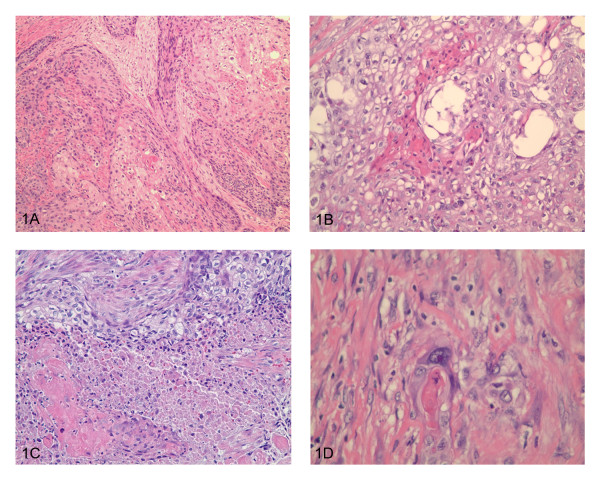
**1A) Infiltrating tumor mass forming nests and cords**. **1B) **Large, cohesive cells, with abundant glassy eosinophilic cytoplasm and well defined cell borders; focal keratinization. **1C) **Focal tumor necrosis. **1D) **Poorly differentiated cells with high grade nuclei infiltrating singly into the stroma. To view the virtual glass slide image for this figure please see here http://diagnosticpathology.slidepath.com/webViewer.php?snapshotId=1304063584.

**Figure 2:**
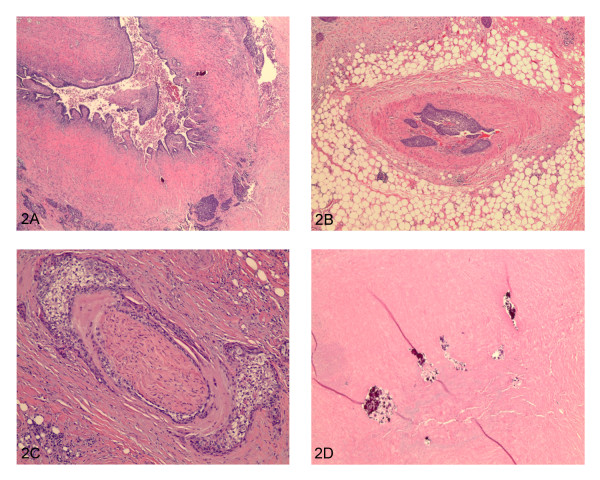
**2A) Tumor extending into seminal vesicles**. **2B) **Lymphovascular invasion. **2C) **Perineural invasion. **2D) **Areas of necrosis with calcifications consistent with therapy effect. To view the virtual glass slide image for this figure please see here http://diagnosticpathology.slidepath.com/webViewer.php?snapshotId=1304063622.

**Figure 3:**
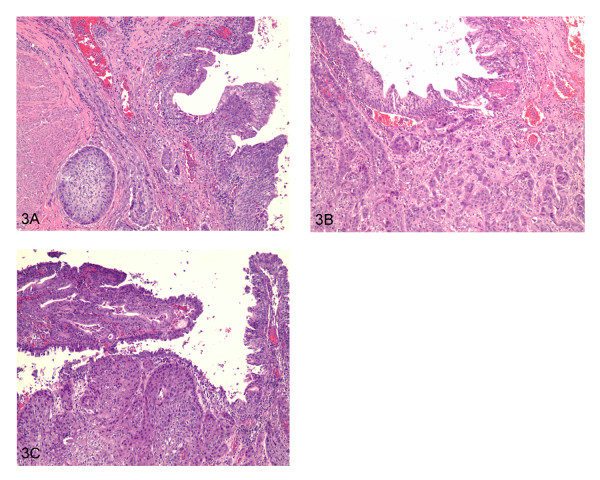
**3A) Hyperplastic urothelium with focal frond-like proliferation**. The urothelial cells were slightly enlarged but the nuclear to cytoplasmic ratio was maintained and the nuclear polarity was preserved. **3B) **Squamous cell carcinoma infiltrating lamina propria and detrusor muscle. **3C) **Squamous cell carcinoma extending, focally, into the urothelial mucosa.

**Figure 4:**
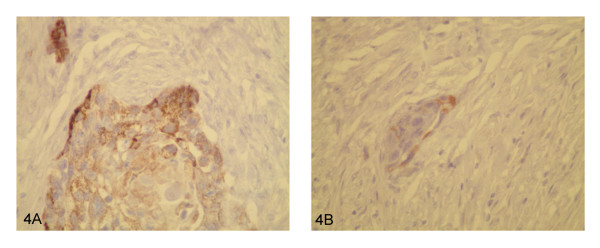
**4A) Viable neoplastic cells staining positive for AMACR**. **4B) **Endothelial cells in the vessels adjacent to the tumor showing focal nuclear AMACR positivity.

Microscopic sections from the prostate showed an infiltrating tumor mass forming nests and cords (Figure 1A). No benign prostatic glands were identified. Cytologically, the cells were large, cohesive, with abundant glassy eosinophilic cytoplasm and well defined cell borders. Keratinization was noted (Figure 1B). The tumor showed focal necrosis (Figure 1C). Areas with less differentiated cells with high grade nuclei infiltrating singly into the stroma were identified (Figure 1D). The morphological appearance was compatible with poorly differentiated squamous cell carcinoma. No other differentiation (adenocarcinoma or urothelial carcinoma) was seen. The tumor replaced the entire prostate, extending into seminal vesicles, periprostatic and perivesicle soft tissue (Figure 2A). Lymphovascular (Figure 2B) and perineural (Figure 2C) invasion were present. There were also areas of necrosis with calcifications consistent with therapy effect (Figure 2D). Sections from the urinary bladder wall revealed inflamed and mainly denuded transitional mucosa with underlying granulation tissue. Where present, the urothelium was hyperplastic with focal frond-like proliferation. Although, cytologically, the urothelial cells were slightly enlarged, the nuclear to cytoplasmic ratio was maintained and the nuclear polarity was preserved (Figure 3A). This degree of atypia was less than that of the tumor cells and was considered to represent reactive changes, secondary to the inflammatory process. No areas of high grade urothelial carcinoma were identified. The lamina propria and detrusor muscle of the bladder were infiltrated by the squamous cell carcinoma (Figure 3B) that also extended, focally, into the urothelial mucosa (Figure 3C). Immunohistochemistry studies, using a battery of prostate specific antibodies, showed viable neoplastic cells staining positive for racemace/AMACR (Figure 4A) and negative for Prostate specific antigen (PSA), Prostate specific membrane antigen (PSMA), Prostate specific acid phosphatase (PSAP) and P501S (553-amino acid protein that is localized to the Golgi complex and expressed in both benign and neoplastic prostate tissues). Interestingly, AMACR also stained the endothelial cells in the vessels adjacent to the tumor (Figure 4B).

A final diagnosis of squamous cell carcinoma involving the prostate and urinary bladder was rendered. The question that arose was whether the squamous carcinoma developed through divergent differentiation from prostatic adenocarcinoma following treatment, represented squamous differentiation of an urothelial carcinoma, or it was the second primary prostatic malignancy in this patient.

## Discussions

Squamous cell carcinoma of the prostate is a rare entity, with an incidence of 0.6-1% of all prostatic malignancies and its etiology is not yet well understood. In about 50% of the cases it arises in the settings of previous radiation or hormonal treatment for prostatic adenocarcinoma but also occurs in the absence of prior treatment. It is believed that the squamous component develops from squamous metaplasia of acini and ductal elements. Non-neoplastic squamous metaplasia is frequently seen in the prostate associated with chronic inflammation or infarction. In addition, malignancies such as hormonal or radiation-treated prostatic adenocarcinoma or urothelial carcinoma can show extensive squamous metaplasia. It has also been hypothesized that it could derive from pluripotent stem cells capable of multidirectional differentiation.

Mott proposed strict criteria for the diagnosis of pure primary squamous cell carcinoma: (1): clearly malignant features including disorganized growth pattern, cellular anaplasia, invasion; (2): features of squamous differentiation including keratinization, presence of squamous pearls or distinct intercellular bridges; (3): lack of glandular/acinar component; (4): no prior estrogen therapy; (5): the absence of primary squamous cancer elsewhere.

We identified in the English literature 66 cases of prostate cancer with squamous differentiation. We initially classified them as either pure squamous cell carcinoma or adenosquamous carcinoma and then each of these two categories was subdivided into cases occurring de novo and cases following therapy for a previously diagnosed prostatic adenocarcinoma (Table 1). Our review of the literature showed that the squamous component was associated with an adenocarcinoma component in 60% of the patients (39 cases, columns one and two). There were 27 patients with pure prostatic squamous cell carcinoma (columns three and four). In about 50% of the cases, the squamous differentiation occurred in patients that were previously treated for prostatic adenocarcinoma (35 cases, columns one and three). In the remaining 31 cases (columns two and four), squamous carcinoma occurred de novo. Within the cases with prior diagnostic of adenocarcinoma, there were 28 patients with adenosquamous carcinoma (column one) but only 7 (column three) with pure squamous carcinoma.

**Table 1:** Classification of patients with squamous cell carcinoma involving the prostate

	Adenosquamous carcinoma		Pure squamous cell carcinoma	
	
	After treatment of adenocarcinoma	De novo	After treatment of adenocarcinoma	De novo
	
	18 cases, Parwani [1]	7 cases, Parwani [1]	3 cases, Parwani [1]	6 cases, Kanthan [10]
	
	6 cases, Bassler [9]	4 cases, Bassler [9]	1 case, Miller [11]	5 cases, Parwani [1]
	
	2 cases, Wernert [12]		1 case, Mohan [3]	2 cases, Nabi [2]
	
	1 case, Braslis [13]		1 case, John TT [14]	2 cases, Little [15]
	
	1 case, Devaney [8]		1 case, Rahmanou [4]	1 case, Wernert [12]
	
				1 case, Okada [5]
	
				1 case, Sarma [6]
	
				1 case, Uchibayashi [16]
	
				1 case, Di Pietro [17]
**Total**	**28 cases**	**11 cases**	**7 cases**	**20 cases**

In most of the cases the diagnosis was made on biopsy or TURP specimens and the criteria set by Mott were followed. In the series of 33 cases of prostate adenocarcinoma with squamous differentiation reported by Parwani [1], there were 8 cases of pure squamous carcinoma (3 cases occurring after treatment of adenocarcinoma and 5 "de novo" cases). To rule out another urological primary malignancy, in all these patients, the bladder was examined cystoscopically and in addition, in one instance, cysprostatectomy was performed which showed no evidence of bladder tumor (Table 2). Nabi [2], Mohan [3] and Rahmanou [4] also excluded another source for squamous cell carcinoma involving the prostate based on a normal cystoscopy exam (Table 2). The patient reported by Okada [5] underwent transurethral biopsy with no evidence of vesicle or urethral malignancy (Table 2). In addition to the case presented by Parwani [1], we identified just one more situation, described by Sarma [6], in which the diagnosis was made after cystprostatectomy procedure. Similar to our patient, no definite site of origin such as urethra or periurethral ducts could be ascertained. Grossly, the bladder mucosa was intact and microscopic sections showed neoplastic cells in the subepithelial tissue with normal overlying urothelium. In conclusion, most of the authors assumed a prostatic origin based on a normal cystoscopy exam. If cystectomy was performed, lack of alterations (in situ or invasive carcinoma) in the transitional lining of the urinary bladder or prostatic urethra, as well as absence of continuity between the squamous neoplastic component and the urothelial mucosa, suggested the prostate as being the source of carcinoma.

**Table 2:** Specimen type and diagnostic modalities to exclude other urological origin in patients with pure squamous cell carcinoma involving the prostate

Reference	Number of patients	Specimen type	Procedures to exclude other urological origin
**Parwani **[1]	8	Prostate biopsy/TURP/prostatectomy/cystprostatectomy	• Normal cystoscopy for 7 patients
			• Cystprostatectomy for 1 patient, with no gross or microscopic evidence of bladder tumor

**Kanthan **[10]	6	Prostate biopsy/TURP	None

**Nabi **[2]	2	Prostate biopsy/TURP	Normal cystoscopy in both patients

**Mohan **[3]	1	TURP	Normal urethroscopy, and cystoscopy

**John TT **[14]	1	TURP	None

**Rahmanou **[4]	1	Prostate biopsy	Normal cystoscopy

**Okada **[5]	1	Prostate biopsy	Transurethral biopsy with no evidence of vesicle or urethral malignancy

**Sarma **[6]	1	Cystprostatectomy	Cystprostatectomy with no gross or microscopic evidence of bladder tumor

However, even after the pathologist recognizes the prostate as the organ of origin, it is still unclear if prostatic squamous carcinoma represents a de novo malignancy or develops from adenocarcinoma following treatment. In our literature review, we found 35 patients treated hormonally or with radiation therapy for prostatic adenocarcinoma that developed subsequently a squamous component (columns 1 and 3 of table 1). Seven of them had pure squamous carcinoma (20%), whereas in the remaining 28 cases (80%), the squamous component was associated with classic adenocarcinoma (adenosquamous carcinoma). This bias suggests that patients treated for previous pure prostatic adenocarcinoma have a propensity to develop squamous differentiation in association with adenocarcinoma and the squamous component represents most probably divergent differentiation of the adenocarcinoma under treatment pressure rather than a second primary prostatic malignancy.

Regardless of its origin, the symptoms of squamous carcinoma of the prostate are usually those of prostatism due to outlet obstruction. The average age of onset is 68 years, ranging from 42 to 86. In those cases where the squamous carcinoma followed radiation or hormonal treatment for adenocarcinoma, the time elapsed varies from 3 months up to 10 years. Our patient developed squamous cell carcinoma 10 years post radiation. Clinical features pointing towards the diagnosis of squamous cell carcinoma include low serum PSA and acid phosphatase levels, high level of squamous carcinoma antigen and osteolytic bone metastasis. Histologically, the degree of differentiation is moderate in most of the cases. If admixed with adenocarcinoma, the squamous component ranges from 5 to 95%. Gleason grading system is not used for squamous component. The morphology (adenosquamous versus pure squamous cell) does not have any prognostic significance.

In many of reported cases, tumor cells stained positive for high molecular cytokeratin (34betaE12) and were negative for PSA and PSAP. We performed immunohistochemistry studies using multiple prostate specific antibodies. The rationale was to identify if the tumor cells in the poorly differentiated areas, that could not be readily classified as squamous or adenocarcinoma, retained the phenotype of prostatic glandular cells with positive staining for any of the antibodies that were used. In concordance with prior reports, the neoplastic cells were negative for PSA, PSAP, PSMA, P501S but showed cytoplasmic staining with AMACR. This immunohistochemistry profile is however not useful for diagnosis since the tumor cells, despite the fact that they have prostatic origin, lose the reactivity for prostate specific antibodies due to their squamous differentiation. The positivity for AMACR is also not helpful because this marker has been reported to be positive in urothelial carcinoma, which could exhibit squamous differentiation as well.

Surprisingly, endothelial cells also demonstrated weak racemase reactivity. Wei Li [7] proved that high AMACR in the cytoplasm of hepatocellular carcinoma tumor cells was significantly associated with venous invasion, suggesting an important role of this enzyme in tumor invasiveness. The exact mechanism of venous invasion remains unclear, but active neovascularization is likely to play an important role.

Molecular studies showed that squamous malignant cells can be either diploid (one case of adenosquamous carcinoma reported by Devaney [8], with well differentiated squamous component), or aneuploid or tetraploid (one case of adenosquamous carcinoma reported by Bassler [9] with moderately differentiated squamous component). This disparity is probably related to the degree of differentiation.

Squamous cell carcinoma of the prostate has a worse prognosis than conventional adenocarcinoma, with an average survival after diagnosis of 1 to 24 months. Therapeutic modalities are limited, and surgery or non-operative methods of treatment (radiation, chemo or hormonal therapy) proved to be ineffective. The resistance of the neoplasm to anti-androgen drugs is well documented and goes along with an origin from the pluripotent cells lining the prostatic ducts. A better understanding of the biology of this tumor might help in the development of more efficient therapy.

## Conclusions

Our patient is the eighth case of pure squamous carcinoma occurring in a patient treated for prior prostatic adenocarcinoma. These cases are the most challenging since oftentimes large tumor volume replaces architectural landmarks and it is difficult to decide if the tumor originates from the prostate or it extends from the urinary bladder into the periurethral/bladder neck tissue. The diagnosis is also complicated by the fact that no residual adenocarcinoma is seen and sometimes even benign prostatic glands are scant or absent due to prior TURP procedure.

**Figure 5:**
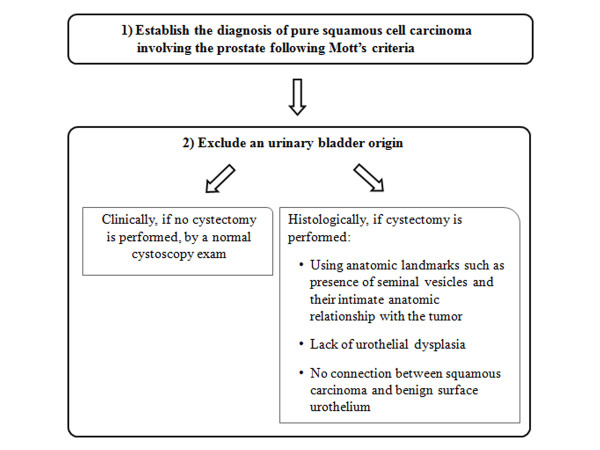
**Our proposed algorithm for work-up of squamous cell carcinoma involving the prostate**.

In these instances we proposed the following algorithm in the work-up of similar cases (Figure 5).

## Consent

Written informed consent was obtained from the patient for publication of this case report and accompanying images. A copy of the written consent is available for review by the Editor-in-Chief of this journal.

## Competing interests

The authors declare that they have no competing interests.

## Authors' contributions

NA has made substantial contributions in acquisition of data, statistical analysis and drafting the manuscript. KD has been involved in revising the manuscript critically for important intellectual content and has given final approval of the version to be published. Both authors have read and approved the final manuscript.
